# Atraumatic Vertebral Dissection in a Patient With Altered Mental Status

**DOI:** 10.7759/cureus.36998

**Published:** 2023-04-01

**Authors:** Tina Kana, Dena Alsurakhi, Ahmed Kawamj, Micheal J Akerman, Nirav Patel

**Affiliations:** 1 Internal Medicine, Touro College of Osteopathic Medicine, New York, USA; 2 Internal Medicine, New York Medical College, Passaic, USA; 3 Pulmonary and Critical Care, New York Medical College, Passaic, USA; 4 Anesthesiology, Touro College of Osteopathic Medicine, Vallejo, USA

**Keywords:** neck pain, headache, nonspecific symptoms, altered mental status, atraumatic vertebral dissection

## Abstract

The most common manifestations of vertebral artery dissection in approximately 80% of patients are headaches or neck pain. We discuss a case of a 34-year-old patient who presented to the emergency department with altered mental status and nonspecific symptoms. A CT angiogram with intravenous contrast revealed a dissection of the left vertebral artery, and the patient was found to have thromboembolism in the right occipital lobe with ischemia on MRI. This case demonstrates the importance of maintaining a broad differential diagnosis for patients who present with altered mental status and nonspecific symptoms such as headache and neck pain in order to adequately diagnose a potentially fatal condition.

## Introduction

Compromised blood flow or thromboembolism is a possible fatal consequence of vertebral artery dissections. Although rare, vertebral artery dissections are a leading cause of stroke in young, healthy, and immunocompetent patients, especially those under the age of 45 years, with a 10% mortality rate and a 20% non-complete recovery rate [1]. The signs and symptoms are often vague and the diagnosis can be ambiguous. Some patients may present with neurological deficits, while others may be totally asymptomatic. 

Dissection of the vertebral artery is caused by a tear in the intima or a rupture of the vasa vasorum, which leads to the separation of the vessel wall layers and the formation of a false lumen [2]. Patients are at an increased risk if they have pre-existing conditions such as connective tissue disorders, with one of the most common being Ehlers-Danlos syndrome [3]. Most vertebral artery dissections, however, result from minor trauma involving neck manipulation or can occur spontaneously. The diagnosis can be challenging, and a thorough history and neurological examination are essential for reaching a proper diagnosis.

## Case presentation

A 34-year-old female patient with a past medical history significant for hypertension, attention-deficit/hyperactivity disorder (ADHD), and anxiety disorder initially presented to the emergency department with slurred speech and an altered mental status. She arrived with her husband who stated that his wife was confused and unable to recall the events that had occurred that morning. According to him, she was generally feeling unwell, and her blood pressure measurement taken at home had shown values of 170/120 mmHg; he also reported that she had an associated headache, neck discomfort, nausea, and vomiting. The patient did not have chest pain, abdominal pain, fever, visual disturbances, weakness, numbness or tingling in the extremities, trauma, or any similar previous episodes as reported by her husband. Following a thorough history, the patient disclosed that renal imaging had been performed as an outpatient, which had suggested a kidney aneurysm. Further questioning revealed that her outpatient prescription medications for hypertension included labetalol and amlodipine, but she was not compliant with them. She had also been prescribed a dextroamphetamine/amphetamine combination product (Adderall) by her psychiatrist but the medication had been recently changed to guanfacine. On examination, the patient was afebrile and her vital signs were within normal limits. A detailed neurologic examination revealed no gross deficits and no facial asymmetry. Additionally, the patient had no carotid bruits and normal visual acuity. The patient appeared ill and somnolent on the physical exam. Although the patient would awaken when spoken to, she continued to alternate abruptly between a placid, subdued mood and one of anxiety, crying loudly. She was not able to follow commands well but appeared to be moving all extremities voluntarily and fully. She appeared to be in mild to moderate distress.

Due to the patient's young age and altered mental status, a comprehensive evaluation was initiated. EKG results revealed sinus tachycardia with a heart rate of 111 bpm and minimal ST depression. Urine toxicity returned positive for amphetamines and negative for ethanol. Complete blood count (CBC) and comprehensive metabolic panel (CMP) were significant for an elevated white blood cell count of 14,100 and a decreased potassium level of 3.2 mEq/L. Other routine lab tests including troponins, international normalized ratio (INR), and partial thromboplastin time (PTT) were reassuring. CT imaging of the head without contrast was unremarkable and showed no evidence of mass or midline shifts. Additionally, no intra- or extra-axial fluid collection was noted. The patient was started on acetylsalicylic acid (ASA, 324 mg) and clopidogrel (300 mg), and those medications were continued at 81 mg and 75 mg respectively. The transient dysarthria eventually appeared to have resolved completely. The patient was subsequently admitted to the hospital and a CT angiogram and additional labs were ordered. 

**Figure 1:**
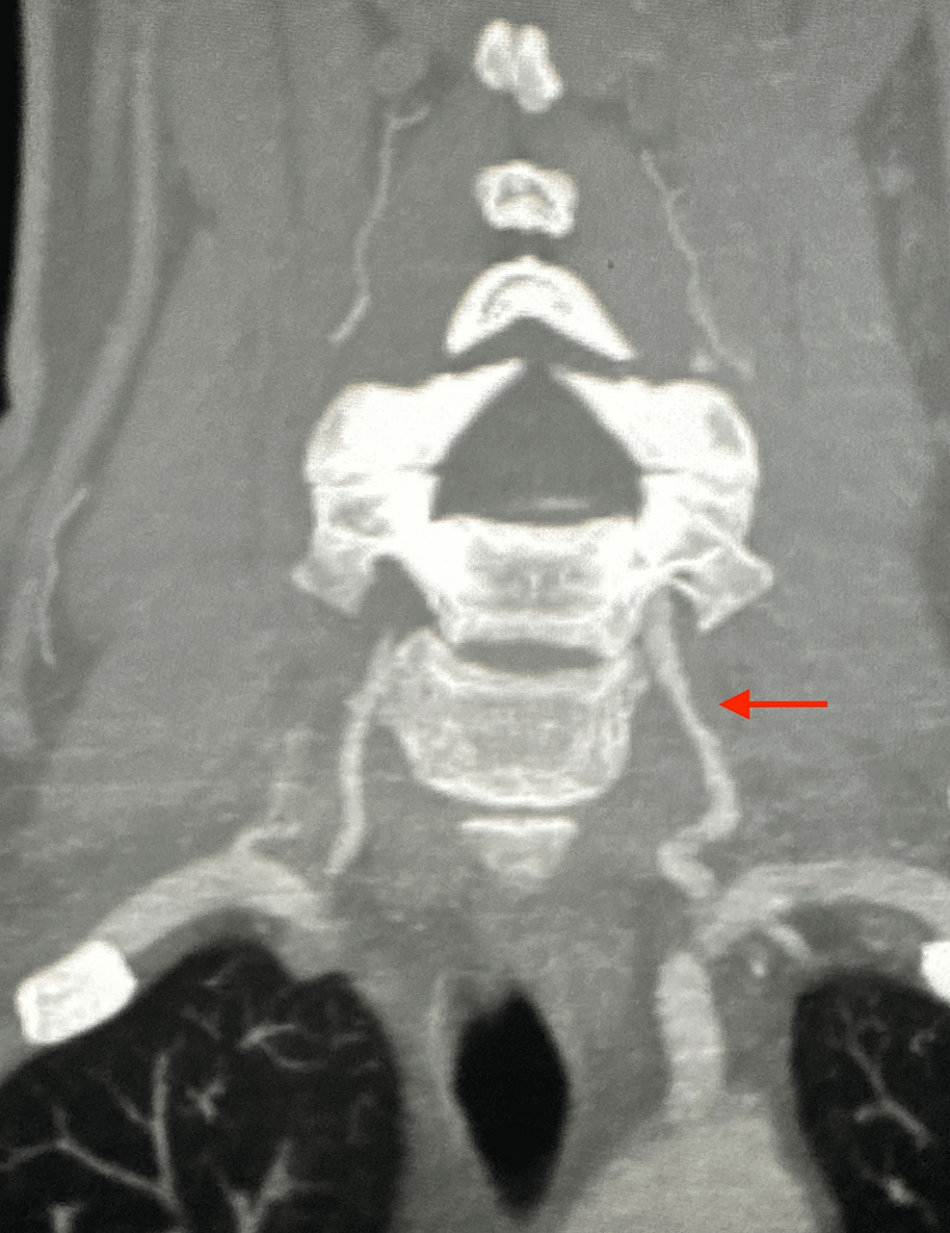
Narrowing and irregularity of the proximal left vertebral artery indicating a dissection

**Figure 2:**
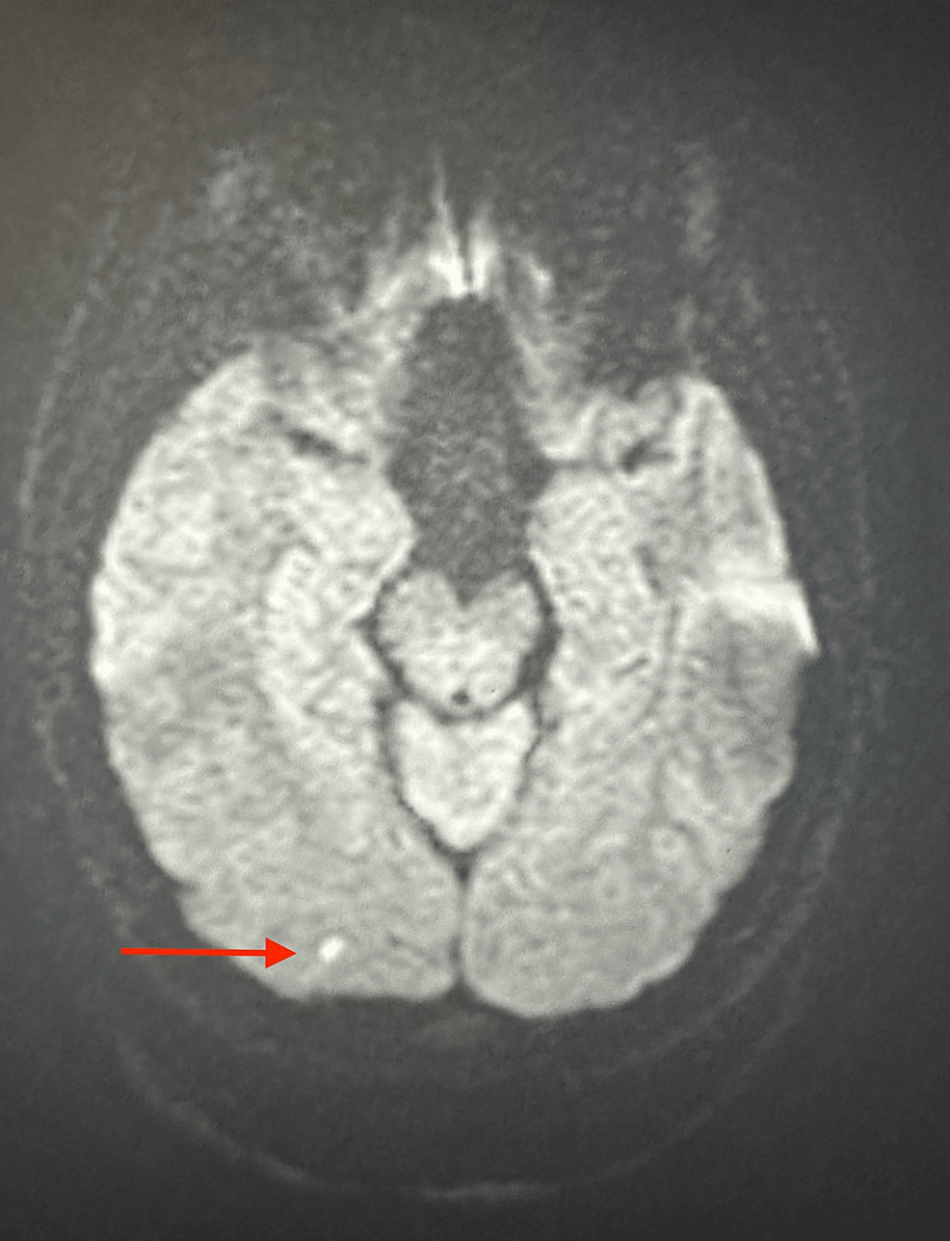
Minute focus of restricted diffusion in the right occipital lobe

CT angiogram of the head and neck demonstrated no intracranial large vessel occlusion. However, the narrowing and irregularity of the proximal left vertebral artery indicated a dissection (Figure 1). Additionally, an MRI of the brain showed a minute focus of restricted diffusion in the right occipital lobe measuring no greater than 4 mm (Figure 2).

The neurology team evaluated the patient, who continued to complain of a headache. It was determined that there was no role for neurosurgery in this patient and a “migraine cocktail” of Toradol, Benadryl, and ondansetron was prescribed. Furthermore, the interventional neurology team advised holding the amlodipine and allowing for permissive hypertension in this patient. The patient’s mental status improved and she admitted that her headache had mostly resolved but that she was not tolerating Benadryl well. Benadryl was discontinued going forward. A repeat CT angiogram of the head and neck ordered three days after admission found no acute changes. There was stable narrowing and irregularity of the proximal left vertebral artery. The patient’s mental status returned to baseline on the day of discharge and she went home on monotherapy of aspirin 81 mg, and amlodipine 5 mg was restarted. The patient was instructed to follow up with her neurologist, cardiologist, and primary care physician within a week.

## Discussion

Vertebral artery dissection occurs predominantly in adults between the ages of 30 and 50 years and with greater prevalence in female patients with approximately 80% experiencing spontaneous dissections [4]. This case illustrates an important tenet in the physician’s evaluation and management of atypical diagnoses that present similarly to other diseases. Our patient had an acute presentation of altered mental status with nonspecific features. Initially, it appeared that there may have been a component of anxiety disorder, but the rapid fluctuation between states of anxiety and flattened affect made this less likely. An acute medical diagnosis appeared more probable based on the patient's sensory manifestations. High on the differential diagnoses was a cerebrovascular accident; however, there was an absence of focal neurological symptoms and the transient dysarthria appeared to have resolved completely, while the general disorientation persisted making this diagnosis less probable.

A diagnosis of vertebral dissection is often more difficult to make other than in cases of carotid dissection, where Horner’s syndrome or lower cranial nerve palsies can aid in establishing a diagnosis. The presentation of symptoms in vertebral dissection varies greatly depending on the location of the dissection. The symptoms range from being asymptomatic to headache, neck pain, facial pain, cranial nerve palsies, nausea, vomiting, and dizziness [5]. Vertebral artery dissections more commonly present as posterior strokes with vertigo, nausea, vomiting, ataxia, and altered mental status [6]. Pain is often a frequent preceding symptom, especially in traumatic dissections, and in spontaneous dissections, it can be delayed for hours to days [7]. It is usually characterized as severe, dull, and non-throbbing and generally localized in the upper portion of the neck and occiput, ipsilateral to the side of the dissection [8]. In our case, the patient presented with generalized neck pain, which many physicians misinterpret as musculoskeletal in nature. Extracranial arteries are pain-sensitive, and in vertebral artery dissections, this is most likely caused by the excitation of nociceptors within the vessel wall, although the exact mechanism is not fully known [9]. Misdiagnosis is highly probable in patients presenting with neck and occipital headaches especially when symptoms of brainstem ischemia are absent.

The gold standard for diagnosis is angiography. Characteristic findings on angiography include long, irregular luminal stenosis, occlusion of the vertebral artery, and pseudoaneurysm [10]. Stenosis, however, is the most common finding in 78% of spontaneous dissections, which is caused by subintimal hematomas [11]. More than 80% of patients will develop posterior circulation ischemia [12]. Transient ischemic attacks and strokes occur by embolism from the dissected artery or by hemodynamic impairment of the area supplied by the occluded or severely stenosed vertebral artery. The MRI of the patient’s brain in our case report showed a minute focus of restricted diffusion in the right occipital lobe. The patient in this case most likely had thromboembolism from the left vertebral artery dissection, which caused a stroke and acute ischemia.

Patients who survive the initial dissection have a good prognosis. Anticoagulation should be started as soon as possible to prevent a stroke [13]. The most common agent used is heparin, which is often transitioned to warfarin, although antiplatelet therapy with clopidogrel and aspirin is equally effective [14]. However, there is no consensus in the literature on the duration of anticoagulation therapy. Repeat imaging in three to six months should be considered to assess the status of the artery or arteries affected by the dissection. For patients treated with anticoagulation in the acute phase, it is reasonable to stop warfarin and start long-term antiplatelet therapy after six months of anticoagulation, as long as symptoms are not recurrent and the arterial lesion is thrombosed or healed.

## Conclusions

While vertebral artery dissection is a rare cause of stroke in general, it represents one of the most common causes in patients younger than 45 years of age. Its signs and symptoms can be ambiguous, and nonspecific symptoms such as headache or neck pain may precede the development of neurological symptoms by as much as two weeks. The main obstacle faced by physicians is that when the workup is initiated for presenting symptoms of headache or neck pain, CT scans and lab results will often be negative. Hence, patients are often misdiagnosed, which delays a proper diagnosis and treatment. A broad differential should be maintained for these patients, and more studies should be conducted to look for an alternative method to avoid these misdiagnoses.
